# Diagnostic Utility of Mast Cell Density and Neutrophilic Infiltration for Differentiating Aseptic Loosening From Periprosthetic Joint Infection: A Retrospective Cohort Study

**DOI:** 10.7759/cureus.103612

**Published:** 2026-02-14

**Authors:** Rameez Ahmedkhan R. Pathan, Akanksha Wankhade, Sujata R Panda, Sandesh Subhash Agrawal, Shahzad Khan

**Affiliations:** 1 Orthopedics, Shri Balaji Institute of Medical Science, Raipur, IND; 2 Orthopedics, Hindu Rao Hospital and North Delhi Municipal Corporation (NDMC) Medical College, Delhi, IND; 3 Orthopedics, SPARSH Hospital, Bengaluru, IND; 4 Orthopedics, Manipal Academy of Higher Education, Mangaluru, IND; 5 Pathology, Shri Balaji Institute of Medical Science, Raipur, IND; 6 Spine Surgery, Shree Narayana Hospital, Raipur, IND; 7 Orthopedics, Sri Devaraj Urs Academy of Higher Education and Research, Kolar, IND; 8 Spine Surgery, Stavya Spine Hospital and Research Institute, Ahmedabad, IND

**Keywords:** aseptic loosening, giemsa stain, histology, mast cells, neutrophils, periprosthetic tissue, prosthetic joint infection

## Abstract

Background

Accurate differentiation between periprosthetic joint infection (PJI) and aseptic loosening (AL) is essential for appropriate surgical planning and optimal postoperative outcomes. Although neutrophil infiltration remains the standard histological marker for diagnosing PJI, it has recognized limitations. Mast cells, which play a role in chronic inflammatory responses, may serve as a complementary biomarker. This study evaluated the combined diagnostic utility of Giemsa-stained mast cell density and neutrophil counts in periprosthetic tissue to distinguish PJI from AL.

Methods

This single-center retrospective cohort study included patients who underwent revision arthroplasty between January 2022 and September 2025. PJI was defined according to the 2018 International Consensus Meeting criteria, whereas AL was diagnosed based on negative microbiological cultures and radiographic evidence of loosening. Periprosthetic tissue samples were stained with H&E for neutrophil assessment and Giemsa stain for mast cell identification. Two blinded observers quantified cells across 10 high-power fields (HPFs; 0.237 mm² per field).

Results

A total of 146 revision cases were analyzed, including 48 PJI and 98 AL cases. Median neutrophil counts were significantly higher in the PJI group than in the AL group (26.5 vs. 1.0 cells/HPF; p < 0.001). Conversely, median mast cell density was significantly higher in AL (11.0 vs. 3.5 cells/HPF; p < 0.001). A neutrophil threshold of ≥5 cells/HPF demonstrated 93.8% sensitivity and 91.8% specificity for diagnosing PJI (area under the curve (AUC) = 0.97). A mast cell cutoff of ≥7 cells/HPF showed 86.7% sensitivity and 85.4% specificity for diagnosing AL (AUC = 0.91). The combined sequential algorithm achieved an overall diagnostic accuracy of 93.2%.

Conclusions

Giemsa-stained mast cell density is a novel, cost-effective histological marker elevated in AL. When combined with neutrophil counts, it improves diagnostic accuracy and may serve as a practical adjunct in the routine pathological evaluation of revision arthroplasty specimens.

## Introduction

Periprosthetic joint infection (PJI) and aseptic loosening (AL) are the two most common causes of failure following total joint arthroplasty and require distinctly different management strategies [1]. PJI typically necessitates prolonged antimicrobial therapy and staged revision procedures, whereas AL is generally treated with single-stage mechanical revision. Accurate differentiation between these entities is therefore critical for appropriate surgical decision-making and optimal patient outcomes [2].

Despite advances in microbiological and serological testing, diagnosing PJI remains challenging, particularly in low-grade, chronic, or culture-negative infections, where conventional markers demonstrate reduced sensitivity and specificity [3]. Consequently, histopathological evaluation of intraoperative periprosthetic tissue has become an integral component of contemporary diagnostic algorithms. Quantification of polymorphonuclear neutrophils (PMNs) per high-power field (HPF) is widely accepted, with thresholds such as ≥5 PMNs/HPF demonstrating good diagnostic performance [4]. However, variability in proposed cutoffs, interobserver differences, and reduced reliability in certain infection subtypes underscore the limitations of relying solely on neutrophil counts and highlight the need for additional complementary biomarkers [5].

In contrast, the biological environment of AL is characterized by a chronic foreign-body inflammatory response to wear particles, mediated by macrophages, fibroblasts, and other immune cells that contribute to osteolysis and implant failure [6]. Mast cells, tissue-resident granulocytes containing inflammatory mediators such as histamine, tryptase, and cytokines, play important roles in chronic inflammation, fibrosis, and bone remodeling [7]. Emerging evidence suggests that mast cell activity differs between particle-induced aseptic inflammation and infection-driven responses, raising the possibility that mast cell density may serve as a useful diagnostic discriminator [8]. Importantly, mast cells can be readily identified using simple and inexpensive histochemical stains such as Giemsa, making this approach practical and accessible in routine pathology settings without the need for immunohistochemistry.

Therefore, the present study aimed to evaluate the diagnostic utility of a dual-marker histological strategy combining conventional H&E-based neutrophil quantification with Giemsa-based mast cell density assessment. We hypothesized that this combined, cost-effective approach would improve discrimination between PJI and AL in revision arthroplasty specimens.

## Materials and methods

Study design and setting

This retrospective diagnostic accuracy study was conducted at Shri Balaji Institute of Medical Science, Raipur. Institutional Ethics Committee approval was obtained prior to study initiation (approval SBIMS/IEC/Certi./150/2025, dated September 16, 2025). The requirement for informed consent was waived due to the use of anonymized archival data. The study was reported in accordance with the Standards for Reporting of Diagnostic Accuracy Studies (STARD) guidelines.

Participants

**Figure 1:**
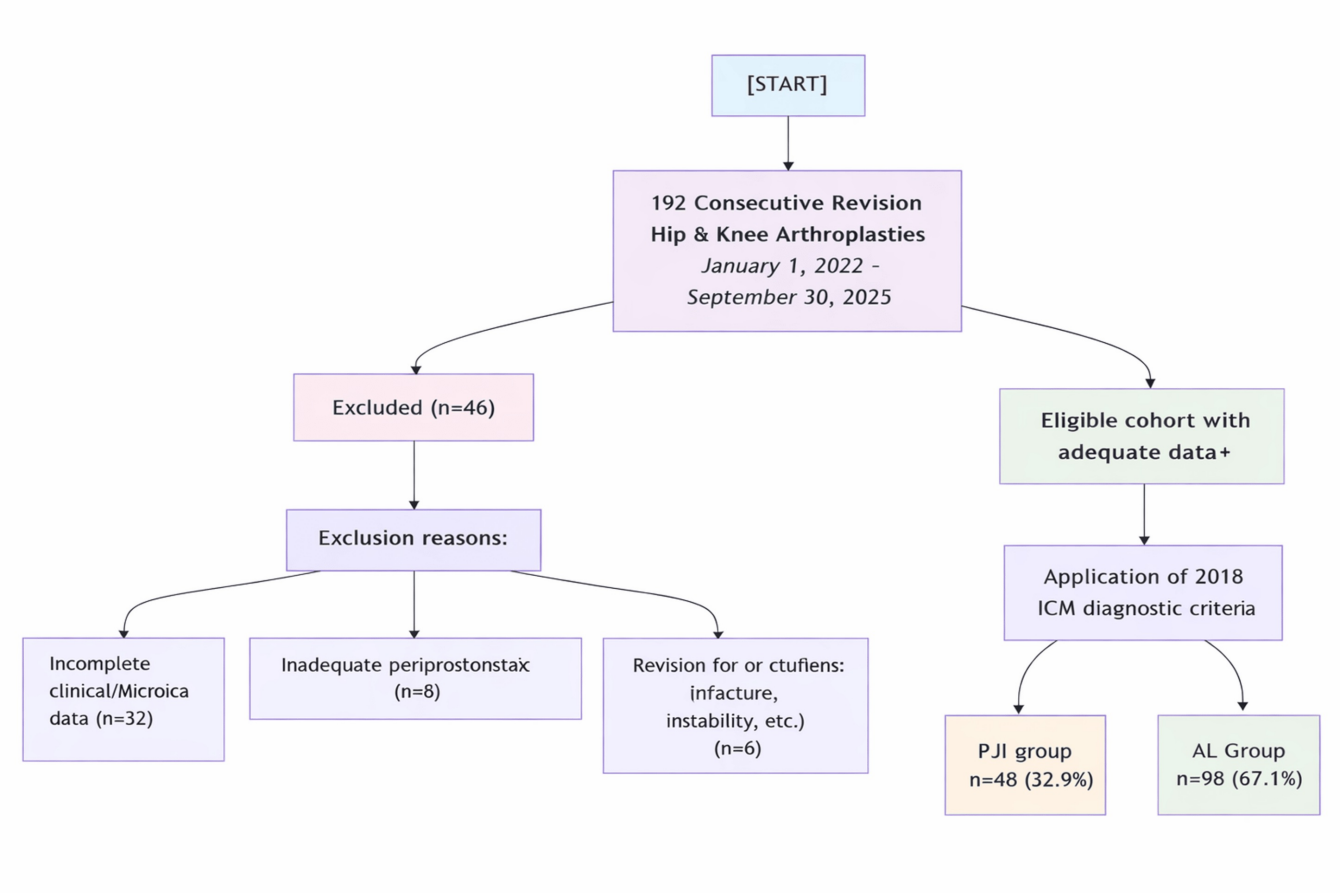
CONSORT-style flow diagram illustrating cohort derivation and application of the 2018 ICM diagnostic criteria AL, aseptic loosening; CONSORT, Consolidated Standards of Reporting Trials; ICM, International Consensus Meeting; PJI, periprosthetic joint infection

Consecutive patients who underwent revision total hip or knee arthroplasty between January 1, 2022, and September 30, 2025, were screened for eligibility. The patient selection process is summarized in Figure 1. Inclusion criteria were revision surgery for suspected AL or PJI, availability of adequate archived periprosthetic tissue samples, and complete clinical, microbiological, and radiological records. Exclusion criteria included inadequate tissue quality, revision for alternative indications (periprosthetic fracture, instability, malalignment, or tumor), insufficient intraoperative microbiological sampling (<3 tissue cultures), and incomplete medical records.

Reference standard and case classification

Cases were classified using a composite reference standard integrating clinical, microbiological, radiological, and intraoperative findings. Patients were assigned to the PJI group if they fulfilled the 2018 International Consensus Meeting (ICM) criteria [9], including the presence of a sinus tract, two positive cultures with phenotypically identical organisms, or a qualifying aggregate diagnostic score. Patients were categorized as AL if no ICM criteria were met, intraoperative cultures were negative (minimum three samples), no clinical signs of infection were present, and radiographs demonstrated mechanical loosening.

Histopathological processing and staining

Formalin-fixed, paraffin-embedded periprosthetic tissue blocks were retrieved from the pathology archive. Two consecutive 4-µm sections were prepared from each case. H&E staining was performed using standard automated protocols for routine assessment. Giemsa staining was performed manually using 10% Giemsa solution prepared in phosphate buffer (pH 6.8), followed by differentiation, dehydration, clearing, and mounting.

Histological quantification and blinding

Slides were independently evaluated by two experienced pathologists blinded to clinical diagnosis and microbiological results. Discrepancies greater than 20% were resolved by joint review to achieve consensus. Cell quantification was performed under 400× magnification using a light microscope (Olympus BX43, Olympus Corporation, Tokyo, Japan), corresponding to an HPF area of 0.237 mm². Ten nonoverlapping HPFs from areas of maximal inflammation were analyzed.

PMNs were counted on H&E-stained sections, and mast cells were identified on Giemsa-stained sections by their characteristic metachromatic cytoplasmic granules. Mast cells were identified using predefined morphological criteria, including round to oval cells with abundant coarse metachromatic purple-blue cytoplasmic granules and centrally to eccentrically placed nuclei. Only intact, clearly delineated cells with visible granules were counted, while fragmented or degranulated cells and staining artifacts were excluded.

**Figure 2:**
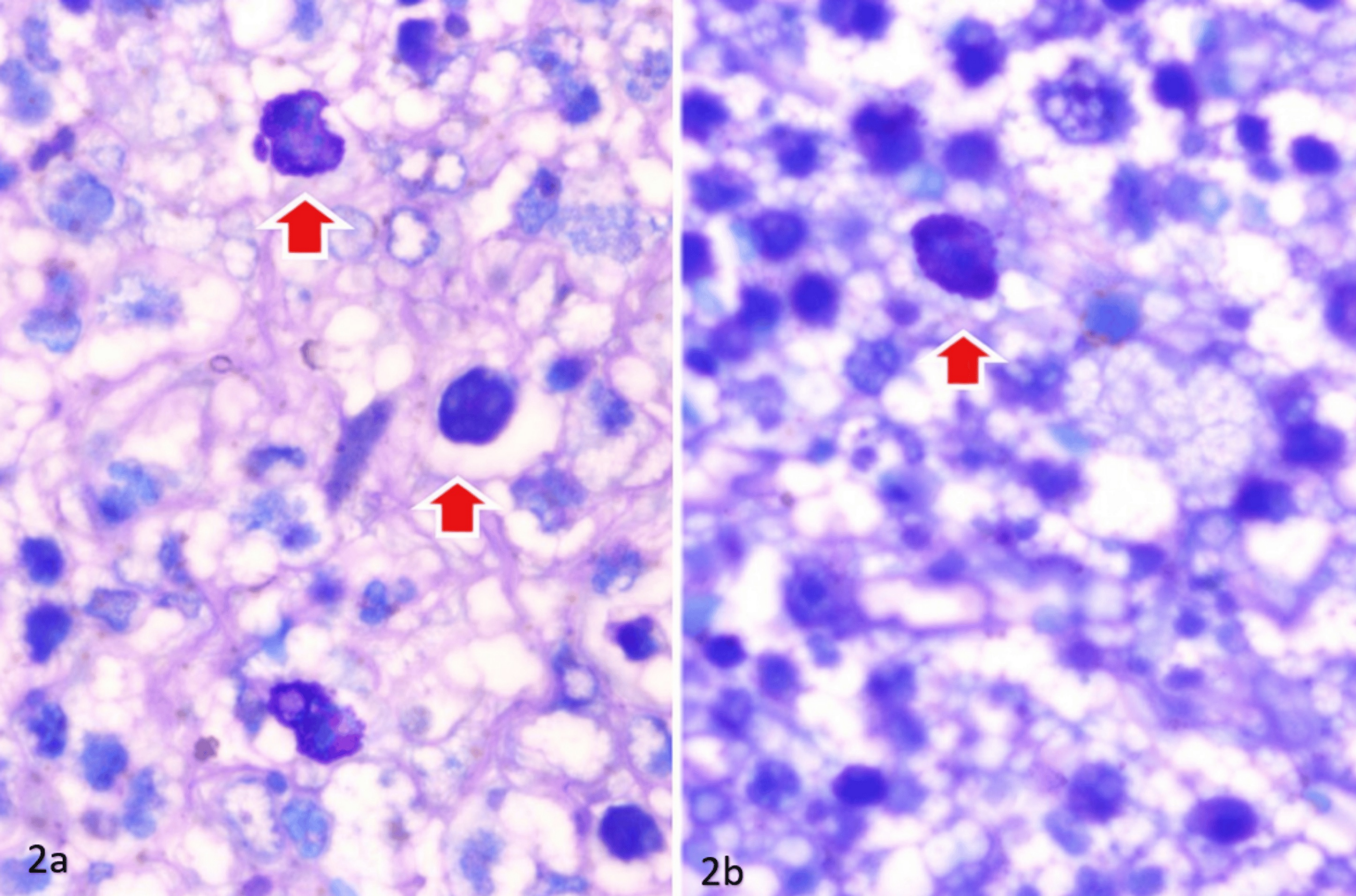
Giemsa stain highlighting the mast cells (red arrows) (a) Tissue section showing mast cells with dense, dark blue-purple cytoplasmic granules highlighted by red arrows on Giemsa staining. (b) Higher magnification/adjacent section demonstrating mast cells with prominent metachromatic granules (red arrow), confirming mast cell infiltration.

Ten nonoverlapping HPFs were selected from areas demonstrating the highest inflammatory cell density while avoiding necrotic tissue, hemorrhage, or section folds. Counts from all fields were averaged to obtain the final value (cells/HPF) for each case. Mean cell counts per HPF were calculated for each case, as shown in Figure 2.

Statistical analysis

Statistical analyses were performed using IBM SPSS Statistics for Windows, Version 26.0 (Released 2018; IBM Corp., Armonk, NY, USA) and MedCalc version 20.0 (MedCalc Software Ltd., Ostend, Belgium). Continuous variables were tested for normality using the Shapiro-Wilk test and are presented as medians with IQRs. Between-group comparisons were conducted using the Mann-Whitney U test.

Receiver operating characteristic (ROC) curve analysis was used to evaluate diagnostic performance. Optimal cutoff values were determined using Youden’s J index. Sensitivity, specificity, positive predictive value, negative predictive value, likelihood ratios, and 95% CIs were calculated. The area under the curve (AUC) was reported.

Interobserver reliability was assessed using the intraclass correlation coefficient (ICC) with a two-way random-effects model. A p-value < 0.05 was considered statistically significant.

## Results

Cohort characteristics

A total of 146 revision arthroplasty cases met the eligibility criteria and were included in the final analysis. Of these, 48 patients (32.9%) were classified as having PJI and 98 patients (67.1%) as having AL. Baseline demographic and clinical characteristics are summarized in Table 1. There were no statistically significant differences between the groups with respect to age, sex distribution, or the joint involved (hip versus knee) (all p > 0.05).

**Table 1:** Baseline demographics and clinical characteristics Values are presented as median (IQR) or number (percentage). Continuous variables were compared using the Mann-Whitney U test, and categorical variables were compared using the chi-square test. AL, aseptic loosening; PJI, periprosthetic joint infection

Characteristic	Total (n = 146)	PJI (n = 48)	AL (n = 98)	p-Value
Age (years), median (IQR)	67.5 (60.0-73.0)	66.0 (58.3-72.0)	68.0 (61.0-74.0)	0.215
Sex, n (%)				0.452
Male	71 (48.6)	25 (52.1)	46 (46.9)	
Female	75 (51.4)	23 (47.9)	52 (53.1)	
Joint revised, n (%)				0.387
Hip	68 (46.6)	20 (41.7)	48 (49.0)	
Knee	78 (53.4)	28 (58.3)	50 (51.0)	

Histological cell counts

Marked differences in inflammatory cell profiles were observed between the two groups. The median neutrophil count was significantly higher in the PJI group compared with the AL group (26.5 vs. 1.0 cells/HPF; p < 0.001). In contrast, mast cell density was significantly higher in the AL group than in the PJI group (11.0 vs. 3.5 cells/HPF; p < 0.001). Detailed distributions are presented in Table 2.

**Table 2:** Comparison of histological cell counts in periprosthetic tissue between PJI and AL groups Cell counts were calculated as the average number of cells across 10 HPFs (0.237 mm² per field). Comparisons between groups were performed using the Mann-Whitney U test. AL, aseptic loosening; HPF, high-power field; PJI, periprosthetic joint infection

Cell type (cells/HPF)	PJI, median (IQR)	AL, median (IQR)	p-Value
Neutrophils	26.5 (14.0-35.3)	1.0 (0.0-3.0)	<0.001
Mast cells	3.5 (1.0-6.0)	11.0 (7.3-15.0)	<0.001

Diagnostic performance of individual markers

**Figure 3:**
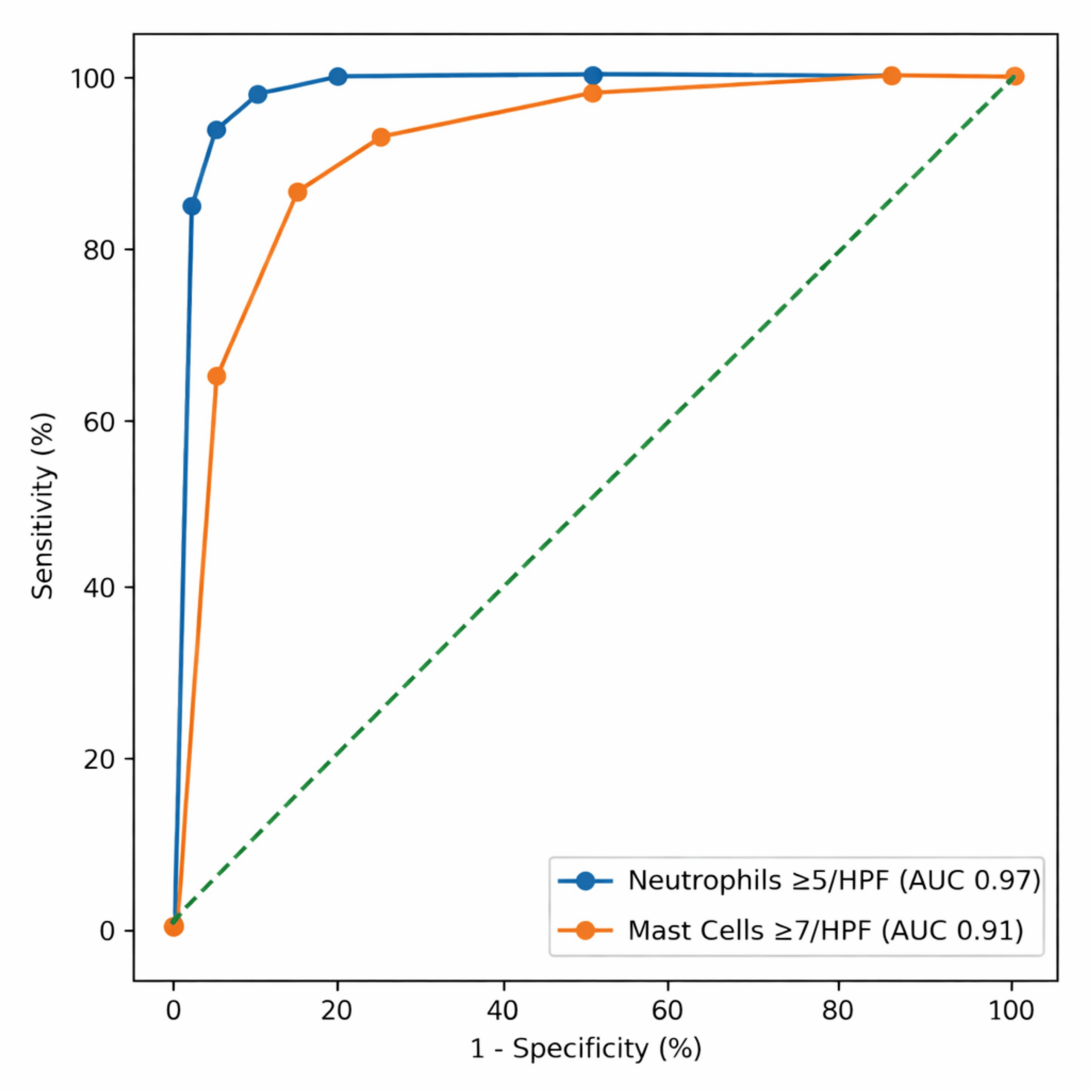
ROC curves for diagnostic parameters AUC, area under the curve; HPF, high-power field; ROC, receiver operating characteristic

ROC curve analysis demonstrated excellent diagnostic performance for both markers (Figure 3). For neutrophils, the established threshold of ≥5 cells/HPF yielded a sensitivity of 93.8% and specificity of 91.8% for diagnosing PJI, with an AUC of 0.97 (95% CI: 0.94-0.99). For mast cells, a cutoff of ≥7 cells/HPF provided optimal discrimination for AL, with a sensitivity of 86.7% and specificity of 85.4% (AUC: 0.91; 95% CI: 0.87-0.95).

Complete diagnostic accuracy metrics, including predictive values and likelihood ratios, are summarized in Table 3.

**Table 3:** Diagnostic performance of histological parameters for differentiating PJI and AL Diagnostic accuracy was assessed using ROC curve analysis. AL, aseptic loosening; AUC, area under the curve; HPF, high-power field; LR, likelihood ratio; NPV, negative predictive value; PJI, periprosthetic joint infection; PPV, positive predictive value; ROC, receiver operating characteristic

Parameter (cutoff)	Sensitivity (95% CI)	Specificity (95% CI)	PPV (%)	NPV (%)	+LR	-LR	AUC (95% CI)
Neutrophils ≥5/HPF (PJI)	93.8 (82.8-98.7)	91.8 (84.5-96.4)	85.7	96.7	11.5	0.07	0.97 (0.94-0.99)
Mast cells ≥7/HPF (AL)	86.7 (78.4-92.7)	85.4 (71.6-94.1)	94	71.2	5.9	0.16	0.91 (0.87-0.95)

Combined diagnostic algorithm

**Figure 4:**
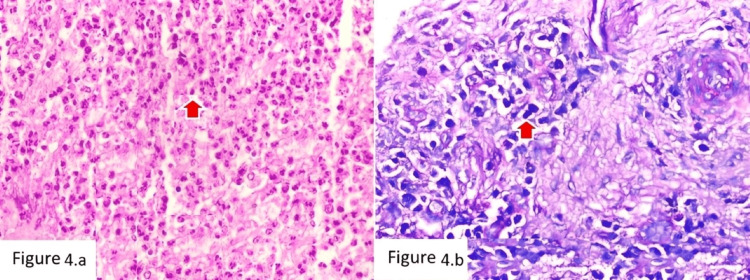
Case of PJI with numerous neutrophils and mast cells (a) H&E stain (×100) showing dense neutrophilic infiltrate with scattered mast cells (red arrow). (b) Giemsa stain (×100) highlighting mast cells with metachromatic cytoplasmic granules (red arrow). PJI, periprosthetic joint infection

**Figure 5:**
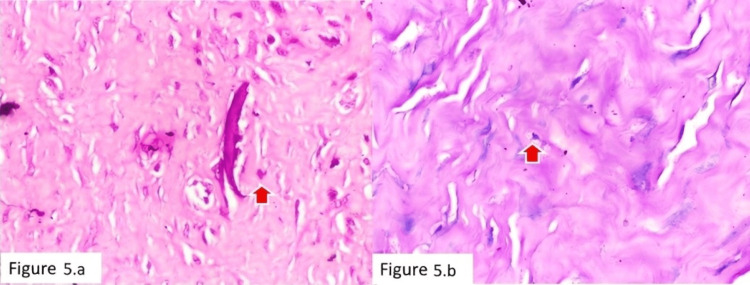
Case of AL with minimal inflammation (a) H&E stain (×100) showing fibrous tissue with sparse inflammatory cells and occasional mast cells (red arrow). (b) Giemsa stain (×100) highlighting mast cells within the fibrous stroma (red arrow). AL, aseptic loosening

A sequential diagnostic approach integrating both markers was evaluated. Applying the neutrophil threshold first (≥5 cells/HPF indicating PJI), followed by mast cell assessment in neutrophil-low cases, correctly classified 136 of 146 cases, corresponding to an overall diagnostic accuracy of 93.2%. Eight cases (5.5%) were negative for both criteria and were categorized as indeterminate. Two illustrative cases are shown in Figure 4 and Figure 5.

Interobserver agreement

Interobserver reliability was excellent for both measurements. The ICC was 0.94 (95% CI: 0.91-0.96) for neutrophil counts and 0.90 (95% CI: 0.86-0.93) for mast cell counts, indicating high reproducibility of the histological quantification method.

## Discussion

Accurate differentiation between PJI and AL remains critical in revision arthroplasty, as treatment strategies and prognoses differ substantially between these entities. In the present study, the established neutrophil threshold (≥5 cells/HPF) demonstrated excellent diagnostic performance for PJI, with an AUC of 0.97, reaffirming the reliability of PMN quantification as a cornerstone of histopathological diagnosis. More importantly, we identified Giemsa-stained mast cell density as a novel and complementary histological marker, significantly elevated in AL and demonstrating robust discriminatory ability (AUC 0.91). When both parameters were applied sequentially, overall diagnostic accuracy exceeded 93%, supporting the value of a dual-marker strategy in routine practice.

The high neutrophil counts observed in infected tissues are consistent with prior literature demonstrating that acute polymorphonuclear infiltration represents the histological hallmark of infection-related inflammation [4,5]. Although serological tests and microbiological cultures remain essential components of diagnostic algorithms, their performance may be reduced in low-grade, chronic, or culture-negative infections [3]. Consequently, intraoperative histology continues to provide critical confirmatory evidence. The sensitivity and specificity achieved in our cohort closely mirror those reported previously, reinforcing the continued clinical utility of this conventional criterion.

In contrast, the significantly increased mast cell density observed in AL provides histopathological support for emerging concepts of aseptic osteolysis as a chronic, particle-mediated inflammatory process rather than an acute infectious response. Periprosthetic membranes in AL are characterized by macrophage-driven foreign-body reactions, fibroblast proliferation, and persistent cytokine signaling that promote fibrosis and bone resorption [6]. Mast cells, as tissue-resident immune cells, contain preformed mediators such as histamine, tryptase, and tumor necrosis factor-α, which are known to influence fibroblast activation, vascular permeability, and osteoclastogenesis [7,10-12]. These mediators may amplify chronic inflammatory pathways and contribute to implant loosening. Conversely, the acute neutrophil-dominant cytokine milieu characteristic of PJI may not favor sustained mast cell recruitment or may lead to rapid degranulation, resulting in lower histological densities [8,13]. This divergence in cellular composition may contribute to the observed diagnostic discrimination; however, given the retrospective observational design, these findings should be interpreted as associative rather than mechanistic.

Recent investigations into the periprosthetic immune microenvironment have similarly highlighted distinct cellular signatures between infectious and aseptic failure, although mast cells have rarely been evaluated specifically [14,15]. Our work contributes directly to this evolving field by quantifying a readily identifiable and reproducible cell population using a simple histochemical technique. Notably, the diagnostic accuracy of mast cell density in our cohort was comparable to that of other proposed adjunctive markers, including specific macrophage or immunophenotypic analyses, but with substantially lower technical complexity and cost [16]. Although several studies have explored histopathological and molecular adjuncts for diagnosing PJI, few have evaluated mast cells specifically as a diagnostic discriminator. To our knowledge, quantitative assessment of mast cell density using routine Giemsa staining has not been systematically investigated in this context, highlighting the incremental and practical contribution of the present study.

From a clinical perspective, the proposed sequential diagnostic algorithm offers a pragmatic and easily implementable workflow. Applying the sensitive neutrophil threshold first allows rapid identification of cases highly suggestive of infection, facilitating timely initiation of antimicrobial therapy and staged revision strategies. Subsequent evaluation of mast cell density in neutrophil-low specimens helps confidently classify a large proportion of AL cases, thereby reducing diagnostic ambiguity. The small proportion of indeterminate cases underscores that histology should serve as an adjunct rather than a replacement for comprehensive clinical, microbiological, and molecular evaluation, particularly in complex or culture-negative presentations [17-20]. Importantly, the excellent interobserver agreement observed in this study indicates that both neutrophil and mast cell quantification are reproducible and suitable for routine pathology reporting.

When compared with other adjunctive diagnostic modalities, this histological approach offers several practical advantages. Techniques such as alpha-defensin assays, leukocyte esterase testing, and next-generation sequencing have demonstrated promising accuracy but may be limited by cost, infrastructure requirements, and availability in many institutions [18-20]. Microbiological cultures are also susceptible to false-negative results due to prior antibiotic exposure or the presence of low-virulence organisms [3,19]. In contrast, histopathological evaluation is universally integrated into revision workflows and provides immediate intraoperative information. Incorporating mast cell assessment into routine microscopy, therefore, represents a straightforward extension of existing practice rather than an additional resource-intensive investigation.

Furthermore, the use of Giemsa staining enhances the translational applicability of this method. Giemsa is inexpensive, widely accessible, and technically simple, making it particularly suitable for resource-constrained or high-volume settings where advanced immunohistochemistry may not be routinely available. Mast cells can be reliably identified by their characteristic metachromatic cytoplasmic granules, without the need for specialized reagents or equipment. This accessibility, combined with the strong reproducibility demonstrated in our study, supports the feasibility of incorporating mast cell density into standardized histopathology protocols and may improve diagnostic confidence across diverse healthcare environments.

Importantly, this study is observational and diagnostic in nature; therefore, differences in mast cell density should be interpreted as associations rather than direct evidence of biological mechanisms. Variations may also reflect sampling heterogeneity, tissue processing factors, or differences in the inflammatory stage. Several limitations should be acknowledged to appropriately contextualize these findings within the existing literature. First, the retrospective single-center design may limit generalizability, and prospective multicenter validation is warranted. Second, although the 2018 ICM criteria serve as an accepted reference standard, misclassification may occur, particularly in culture-negative infections. Third, Giemsa staining identifies mast cells morphologically but does not distinguish subtypes or activation states, which may be better characterized using immunohistochemical markers such as tryptase. Fourth, tissue sampling variability within the periprosthetic membrane may influence cell counts. Finally, correlations between mast cell density, wear particle characteristics, and long-term clinical outcomes were not assessed and represent important directions for future research.

## Conclusions

This study reaffirms the strong diagnostic utility of histopathological neutrophil quantification for identifying PJI. Additionally, it demonstrates that Giemsa-stained mast cell density may serve as a useful, inexpensive, and reproducible adjunctive marker, more frequently observed in AL. The complementary biological patterns of these two cell populations enable improved differentiation between infectious and aseptic causes of arthroplasty failure. Incorporating mast cell assessment alongside routine neutrophil counts within a simple sequential diagnostic algorithm enhances overall diagnostic accuracy and correct classification rates. Given its low cost, technical simplicity, and compatibility with standard staining protocols, this combined histological approach can be readily integrated into routine pathology workflows, particularly in resource-limited settings, providing practical and reliable support for clinical decision-making during revision arthroplasty.
